# Enthalpy Change from Pure Cubic Ice I_c_ to Hexagonal Ice I_h_

**DOI:** 10.1021/acs.jpclett.3c00408

**Published:** 2023-05-25

**Authors:** Christina M. Tonauer, Keishiro Yamashita, Leonardo del Rosso, Milva Celli, Thomas Loerting

**Affiliations:** † Institute of Physical Chemistry, University of Innsbruck, Innrain 52c, A-6020 Innsbruck, Austria; ‡ Consiglio Nazionale delle Ricerche, Istituto di Fisica Applicata ‘Nello Carrara’, via Madonna del Piano 10, I-50019 Sesto Fiorentino, Italy

## Abstract

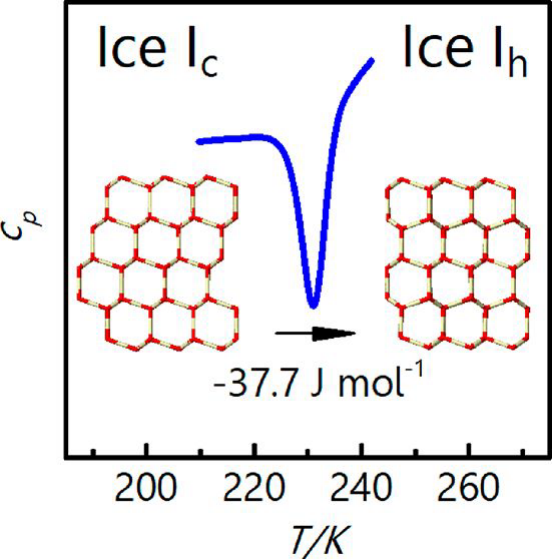

The preparation of pure cubic ice without hexagonal stacking faults has been realized only recently by del Rosso et al. (Nat. Mater.2020, 19, 663−6683201553310.1038/s41563-020-0606-y) and Komatsu et al. (Nat. Commun.2020, 11, 46432015342). With our present calorimetric study on the transition from pure cubic ice to hexagonal ice we are able to clarify the value of the enthalpy change Δ*H*_c→h_ to be −37.7 ± 2.3 J mol^–1^. The transition temperature is identified as 226 K, much higher than in previous work on ice I_sd_. This is due to a catalytic effect of hexagonal faults on the transition, but even more importantly due to a relaxation exotherm that was not properly identified in the past.

Cubic ice (I_c_) was first proposed by König in 1943,^1^ but its first successful experimental preparation was achieved only recently.^2,3^ This metastable polytype of ice I differs from stable hexagonal ice (I_h_) in terms of the stacking arrangement of layers of 6-membered rings of water molecules, which are connected via hydrogen bonds. While hexagonal ice shows an ABAB stacking (where A is a mirror image of B), cubic ice consists of layers in an ABCA arrangement. However, all “cubic ices” studied in the past actually contain significant numbers of hexagonal stacking faults, depending on the mother phase they crystallized from.^4^ In retrospect, all these ices should have been termed stacking-disordered ice (ice I_sd_), but not ice I_c_.^5^ Studying the relative stabilities between the three different ice I polytypes has so far not been possible in experiments due to the unavailability of pure ice I_c_. Especially the enthalpy difference between ice I_c_ and ice I_h_ Δ*H*_c→h_ is not known, but of fundamental interest. While it is settled that ice I_h_ is the stable phase in the bulk at atmospheric conditions, simulations on mW water by Lupi et al. show that, for very small sizes (up to 100,000 molecules), in fact ice I_sd_ is more stable than both ice I_c_ and ice I_h_ at 230 K.^6^ This is rationalized in terms of entropy, specifically entropy gain through stacking-disorder, and supported by experimental work from Mayer & Hallbrucker,^7^ who first showed that ice I_sd_ first nucleates from rapidly cooled liquid droplets at <200 K, before transforming to stable ice I_h_. While there is considerable computational work on thermodynamic stability of the ice I polytypes, utilizing different water potentials (mW,^6,8,9^ ST2,^10^ TIP4P/Ice,^11−13^ TIP4*P*/2005^14,15^) as well as *ab initio* methods,^16,17^ experimental data on the Gibbs free energy, enthalpy, and entropy are notoriously difficult to access. Hondoh et al.^18,19^ approached this question applying X-ray topography to study formation and annihilation of stacking faults and reported a free energy cost of 0.31 mJ m^–2^ (16.5 J mol^–1^ ^6^) at 253 K, associated with the annihilation of a hexagonal stacking fault in ice I_c_.

In terms of enthalpy difference, numerous calorimetric studies are available that have scrutinized the transition from different stacking-disordered ices I_sd_ to I_h_, as listed in Table 1. The reported literature values for the enthalpy change scatter by an order of magnitude, ranging from less than −10 ^20^ to ca. −160 ^21^ J mol^–1^. The difference in the reported results is not only due to the different number of hexagonal stacking faults in differently prepared variants of ice I_sd_. Much more so, there are fundamental issues about the nature of the heat release observed in calorimetry experiments on powdered samples. This becomes obvious by comparing the different calorimetric signatures and peak integration methods. Some studies report the existence of a “pre-peak” around ∼170 K (see Table 1), prior to the polytypic transition exotherm,^22,23^ often assigned to growth of crystallites, crack healing, and strain relief.^24^ Other authors observe only one, “merged” exothermic feature between ∼170–240 K,^21,25−29^ and some authors report both cases, depending on differences in heating rate and powdering of samples.^7,24^ It has remained unclear so far, what actually causes a double-peak and what causes a merged, single peak in calorimetry experiments, where typically very slow heating on the order of K/h was employed in Tian-Calvet calorimetry, but faster heating on the order of K/min in differential scanning calorimetry experiments. It is thus an open question, how to properly integrate the peaks. This question has to be addressed together with the differing cubicity in ice I_sd_,^30,31^ i.e., different numbers of hexagonal stacking faults for differently prepared forms of ice I_sd_. In the present work we make use of a fully cubic sample, so that differing cubicity is no longer an issue, and so that we are able to assign the prepeak to relaxation phenomena unrelated to the actual rearrangement from cubic to hexagonal stacking sequences. That is, with the experimental accessibility of pure cubic ice (crystallized from ice XVII^2^ or hydrogen hydrate^3^), this study is aimed at the open question about the enthalpy difference between ice I_c_ and ice I_h_, Δ*H*_c→h_.

**Table 1:** Comparison of Calorimetry Studies on the Ice I_sd_ to Ice I_h_ Transition^a^

			Pre-Peak	Ice I_sd_ → Ice I_h_	
Study	Mother phase	Heating rate/K min^–1^	*T*_init_; *T*_final_/K	*T*_init_; *T*_final_/K	Δ*H*_sd→h_ /J mol^–1^
Handa et al. (1986)^22^	uHDA	0.167	190; 219	219 ± 2; 232 ± 0	–16 ± 5
Beaumont et al. (1961)^40^	ASW	-	-	∼200	<−106 *
Sugisaki et al.(1968)^21^	ASW	-	merged	160; 210	–161 ± 15 *
McMillan & Los (1965)^26^	ASW	16	-	∼186 (*T*_onset_)	–151 *
Ghormley (1968)^23^	ASW	20	193; 223	223; 268	< −20
Mayer & Hallbrucker (1987)^7^	HGW (190 K)	10	merged	∼223 (*T*_min_)	–56 *
	HGW (170 K)	10	2 subminima	-	–56 *
Johari et al. (1990)^27^	HGW (77 K)	30	merged	224 ± 2 (*T*_min_)	–50 to –60 *
Kohl et al. (2000)^24^	HGW (130 K)	30	∼193 (*T*_min_)	∼230 (*T*_min_)	–40 *
	HGW (140 K)	30	∼193 (*T*_min_)	∼230 (*T*_min_)	–36 *
	HGW (150 K)	30	∼193 (*T*_min_)	∼230 (*T*_min_)	–33
	HGW (160 K)	30	2 subminima	-	–128 *
	HGW (170 K)	30	2 subminima	-	–147 *
	HGW (190 K)	30	merged	-	–96 *
Handa et al. (1986)^28^	ice V	0.167	merged	179 ± 2; 217 ± 1	–51 ± 1 *
	ice VI	0.167	merged	177 ± 1; 217 ± 0	–50 ± 4 *
Yamamuro et al. (1987)^29^	III, IX	0.042	3 subminima	165; 225	–37 ± 1 *
Handa et al. (1988)^25^	II	0.167	merged	186 ± 6; 227 ± 3	–36 ± 4
	IX	0.167	merged	179 ± 4; 230 ± 4	–13 ± 4
	VIII	0.167	merged	186 ± 0; 228 ± 0	–35 ± 1
	LDA	0.167	-	-	–35 ± 4
Salzmann et al. (2004)^41^	IV	5	-	∼216 (*T*_min_)	–20 ± 7
	XII	5	-	∼217 (*T*_min_)	–31 ± 3
Fuentes-Landete et al. (2020)^20^	II	30	-	∼210 (*T*_init_)	<−10

a *T*_init_ and *T*_final_ denote the initial and final temperature of a peak in a calorimetric scan, respectively, i.e., the integration limits for determination of the enthalpy change. *T*_onset_ and *T*_min_ represent the temperatures at the onset and the minimum of an exothermic feature, respectively, as shown in Figure 1a. “Merged” implies that the pre-peak overlaps with the main peak for the ice I_sd_ to ice I_h_ transition. Based on the results of the present study, we mark enthalpy values that were likely overestimated in literature by (*).

In addition to the relevance for thermodynamics of ice I polytypes, this study has further implications for our understanding of fundamental processes in Earth’s atmosphere, e.g., the formation of cold cirrus clouds in the upper troposphere^32^ or noctilucent/polar mesospheric clouds (PMCs) at even higher altitudes in Earth’s mesosphere (∼80 km).^33^ While there has been consensus that ice I_sd_ exists in Earth’s atmosphere in addition to predominant ice I_h_,^34−38^ likely based on preferential formation of small stacking-disordered crystallites via homogeneous nucleation,^6^ a recent study has shown that cubic ice crystallites preferentially form via heterogeneous nucleation on graphene or h-BN at ∼110 K,^39^ a plausible mechanism at mesospheric conditions.

We here present differential scanning calorimetry (DSC) measurements of the cubic to hexagonal ice transition, starting from pure H_2_O cubic ice transformed from XVII, that is, void of hexagonal stacking faults.^2^

Figure 1a depicts one representative heating scan of a sample of ice I_c_ between 160 and 250 K. Figure 1b shows a powder X-ray diffractogram of a sample of cubic ice at 80 K, with sample holder peaks marked by asterisks. The three intense Bragg peaks at 24.19°, 40.02°, and 47.31°, representing the 111, 220, and 311 reflexes, show that the sample under scrutiny is ice I_c_. For stacking-disordered hexagonal ice the 111 peak is usually significantly broadened and shows a shoulder at ∼22.8° which originates from hexagonal stacks. In our case, there is a tiny peak at ∼23.00°, where the intensity is less than 3% of the 111 Bragg peak intensity. Other Bragg peaks of ice I_h_ are below the noise level of the measurement (positions marked by crossed gray arrows, taken from ref (42)), which indicates that no hexagonal ice has condensed from air onto the sample. That is, there is a very small inherent amount of hexagonal stacking faults in our pure cubic ice. For comparison, “cubic ices” studied in the past prepared from other mother phases, e.g., from high-pressure ice polymorphs, from amorphous ice, or from the liquid, feature an intensity of the 100 Bragg peak of ice I_h_ at least an order of magnitude larger than seen in Figure 1b. This is why we follow the literature practice to call this ice “pure” ice I_c_, essentially void of ice I_h_.

**Figure 1:**
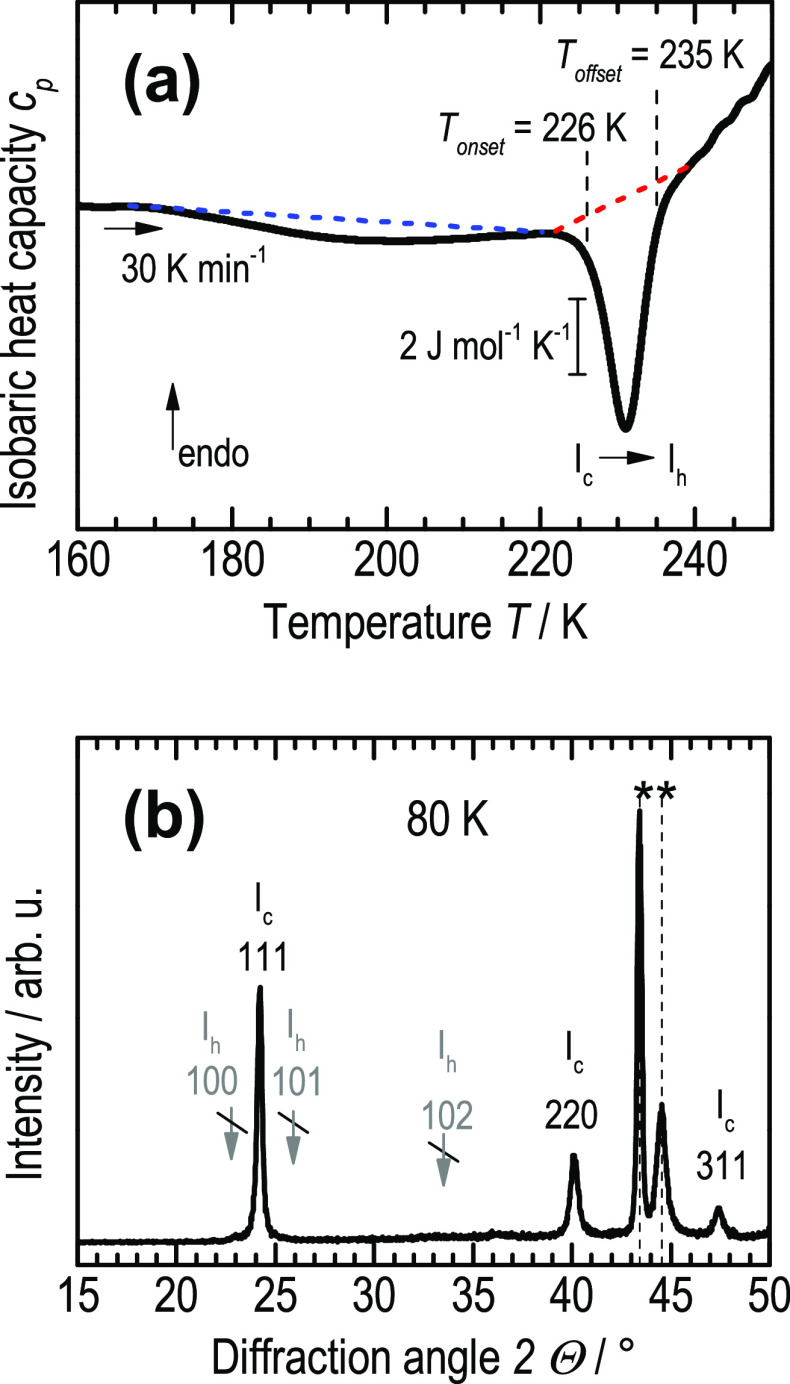
(a) Thermogram *c*_p_(*T*) of a heating scan (after baseline correction) of a sample of pure cubic ice showing the sharp and well-separated I_c_ to I_h_ transition exotherm centered around 231 K. (b) Powder X-ray diffractogram of pure cubic ice at 80 K, showing only 3 characteristic Bragg peaks at 24.19° (3.676 Å), 40.02° (2.251 Å), and 47.31° (1.920 Å) characteristic for ice I_c_.^2^ As a reference, the positions of characteristic Bragg peaks of ice I_h_ at 22.79° (3.899 Å), 25.80° (3.442 Å), and 33.62° (2.670 Å) are marked by crossed gray arrows.^42^ Sample holder peaks are marked by asterisks. Diffraction angles at *x*-axis correspond to Cu Kα radiation.

The thermogram *c*_p_(*T*) in Figure 1a recorded using a heating rate of 30 K min^–1^ clearly shows that the I_c_ to I_h_ transition appears as a sharp exothermic feature centered around 231 K, with an onset temperature *T*_onset_ of 226 K (determined by the intersection of two straight lines extrapolating the baseline and the peak edge of the exotherm, respectively, see SI Table 1). The integration of this peak (within the limits of 221 and 240 K, sketched by the red dashed baseline) yields the enthalpy of transition from cubic to hexagonal ice.
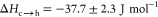
(Average of 9 independent measurements, standard error given as standard deviation based on a sample of the population, see SI Table 1).

We emphasize that the transition starts from hydrogen-disordered ice I_c_ and ends in hydrogen-disordered ice I_h_. That is, there is no entropy difference between the two ices in terms of hydrogen order. Hydrogen ordering in ice I is of relevance only below 72 K.^43^ Our enthalpy difference compares with a calculated difference of 0 ± 30 J mol^–1^ in mW water.^44^ Considering the free energy change Δ*G*_c→h_ = −16.5 J mol^–1^ measured by Hondoh,^18,19^ our result implies that cubic ice is destabilized by enthalpy but stabilized by entropy (which does not originate from Pauling entropy). In addition, we performed DSC scans of ice I_c_ at heating rates of 10 and 50 K min^–1^, successively (see SI Figure 2). Applying different heating rates shows an expected effect on the onset temperatures, where *T*_onset_ increases with increasing heating rate (SI Figure 2) but shows no effect on Δ*H*_c→h_ (see SI Table 1). Also, we measured thermograms starting from a sample of ice XVII as precursor for ice I_c_ (SI Figure 3). While these scans show the same transition enthalpy Δ*H*_c→h_, the I_c_-I_h_ transition is shifted to ∼3 K higher onset temperatures compared to experiments starting from ice I_c_. We interpret this as a result from different thermal history, once again emphasizing the metastable nature of ice I_c_. Specifically, the cubic ice that forms in the course of the DSC heating scan *in statu nascendi* from ice XVII needs a bit more time to rearrange to hexagonal ice compared to pure ice I_c_ prepared outside the DSC instrument.

Furthermore, our heating experiments of cubic ice exhibit a broad and weak exothermic feature prior to the polytypic transition, centered around ∼193 K (Figure 1a). It shows an enthalpy change of −14.4 ± 2.3 J mol^–1^ (integration limits marked by the blue dashed baseline in Figure 1a, for measured values see SI Table 2). This feature has also been reported by Kohl et al. in their calorimetry (and X-ray diffraction) study of hyperquenched glassy water (HGW)/ice I_c_.^24^ They assigned the weak exotherm to a relaxation phenomenon, likely composed of effects from coalescence of particles, relief of nonuniform strain, and healing of different kinds of defects. Also in case of the powder of cubic ice employed here, relaxation (probably at the grain interfaces) takes place prior to the transition itself. As evident in SI Figure 2 this feature is different for different heating rates and furthermore also different depending on whether cubic ice was made beforehand or inside the DSC instrument (compare SI Figures 2 and 3). In other words, the size of this feature depends very much on the size of ice grains and/or morphology but is not related to the actual I_c_ to I_h_ transition. Employing heating rates of 10–50 K min^–1^ allows for clear separation of the relaxation exotherm from the I_c_ to I_h_ transition in the present study. That is, the prepeak is related to the powdering of the sample, size of grains, etc., but unrelated to the inherent rearrangement of cubic to hexagonal stacking sequences.

Considering the literature data shown in Table 1, studies integrating a “merged” exothermic feature overestimate the heat release. That is, all enthalpies that are labeled “merged” in Table 1 contain a contribution from relaxation that is not related to the bulk enthalpy for the cubic-to-hexagonal transition. The “true” Δ*H*_c→h_(ice I_sd_) in these studies is less negative, closer to zero, than the apparent Δ*H*_c→h_(ice I_sd_) reported by the authors. We mark the studies which likely overestimated the enthalpy change by (*) in Table 1. For example, Handa et al.^28^ performed heating scans of different samples of stacking-disordered ice I_sd_, crystallized from ice V and ice VI, respectively, at ∼0.17 K min^–1^ and observed only one exotherm between ∼175 and 220 K. This can be rationalized by the low heating rate and a catalytic effect of the hexagonal stacking faults within the cubic ice sample, lowering the onset temperature of the cubic to hexagonal transition. As a result, the two exothermic events ((i) relaxation and (ii) transformation of ice I_c_ to ice I_h_) merge to one. Therefore, the value of Δ*H*_c→h_ was overestimated to be ca. −50 J mol^–1^,^28^ very close to the sum of the two heats reported here. A similar catalytic effect was also found in a Raman spectroscopy study of the ice I_c_-I_h_ transformation in the same pure cubic ice samples as studied here,^45^ where an even slower heating ramp (namely, 0.037 K min^–1^) was applied, and the transformation was completed at temperatures below 200 K. That is, even the tiny amount of hexagonal stacking faults noted in Figure 1b catalyzes the transition. Furthermore, Yamamuro et al.^29^ likely reported a too-large (negative) enthalpy change, even though their value (−37 ± 1 Jmol^–1^) coincides with the value presented here. However, in light of the present study, their integration limits were chosen too broadly. Considering their Figure 12 of ref (29), integration between ∼200 and ∼230 K, excluding the prepeak feature merged with the actual I_sd_-I_h_ transition exotherm, seems most accurate.

With the present finding of Δ*H*_c→h_ = −37.7 ± 2.3 J mol^–1^ we finally establish clarity for the long-standing inconsistency related to the transformation enthalpy of cubic to hexagonal ice in bulk samples. This result is a benchmark for future simulations aimed at the equilibrium between liquid and crystalline water at ambient pressure.^8,10,14,16^ Furthermore, this result can be applied for an approximation of the difference in vapor pressure between ice I_c_ (*p*_c_) and ice I_h_ (*p*_h_), a key question for supersaturation above ice clouds and the mechanism of cloud formation.^46^ Based on the Clausius–Clapeyron equation, the ratio of the vapor pressures is often approximated^32^ as
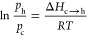
1

Entering the value of Δ*H*_c→h_ into eq 1 shows that the vapor pressure of pure ice I_c_ is only 3% higher than the one of ice I_h_ at 170 K and only 2% higher at 230 K. In terms of absolute vapor pressure of cubic ice, we can then use the vapor pressure reported for ice I_h_ by Marti & Mauersberger^47^ as well as Mauersberger & Krankowsky.^48^ Adding the 3% and 2%, the absolute vapor pressure above ice I_c_ calculates as 7.19 × 10^–4^ Pa at 170 K and 10.2 × 10^–4^ Pa at 230 K. The vapor pressure of ice I_sd_ particles in clouds will inevitably be between the two values, i.e., even closer to the one of ice I_h_. Any form of stacking-disordered ice containing a significant amount of hexagonal stacking faults will inevitably show a less negative Δ*H*_c→h_ and accordingly an even smaller difference in vapor pressure compared to pure hexagonal ice. That is, our result is inconsistent with measurements reporting a difference in vapor pressure of ∼10% between ice I_sd_ and ice I_h_,^32,49^ with the need of clarification and more direct measurements of the vapor pressure of ices I_c_ and I_sd_ in the future. Calorimetric studies providing values more negative than −37.7 ± 2.3 J mol^–1^ are affected by relaxation effects and peak merging without proper separation from the exotherm related to the transition itself (Table 1).

Moreover, the measured onset temperatures of the cubic to hexagonal ice transition of 220, 226, and 230 K at 10, 30, and 50 K min^–1^, respectively, provide important clues for understanding which forms of ice are present in the layers of our atmosphere and how their structure influence properties such as Earth’s albedo.^50−52^ “Cubic ice” in our atmosphere is more stacking-faulty than the ice studied here, so it will experience an even more pronounced catalytic effect of pre-existing hexagonal stacks. For that reason, the transition from ice I_sd_ to ice I_h_ can take place at temperatures below 200 K. On the contrary, the uncatalyzed transition will take place at higher temperature. For pure ice I_c_ made from the C_2_ structure of hydrogen hydrate H_2_–H_2_O (by complete degassing of hydrogen, resulting in the empty cubic host structure as reported by Komatsu et al.^3^) a transition temperature above 240 K was found. It would be very interesting to study a sample prepared in that manner calorimetrically, but so far such samples have not been recovered for ex situ measurements. Knowledge of the phase behavior of the different polytypes of ice I, e.g., their stability with respect to each other combined with remote sensing, will facilitate new insights of the thermal history of celestial bodies.^53^

## Experimental Methods

Pure ice I_c_ was obtained by heating a powder of ice XVII to 150 K. The precursor material ice XVII was previously obtained by means of a suitable thermal treatment of hydrogen hydrate samples in C_0_ structure.^54^ In total we recorded nine heating scans at 10, 30, and 50 K min^–1^ of samples of ∼10–20 mg of cubic ice in aluminum crucibles using a *DSC8000* by PerkinElmer. After the first heating scan, the sample (after transformation to ice I_h_) was cooled down to ∼93 K and reheated in a second heating scan. This second scan (of ice I_h_) was used for baseline correction (see SI Figure 1). The calorimetric features were normalized by the melting enthalpy of ice I_h_, i.e., 6012 J mol^–1^ at 273 K. The uncertainty of measured temperatures is ±1 K (for more detail about the method and instrument calibration, see ref (55)). Powder X-ray diffraction at ∼80 K and subambient pressure (∼1 mbar) using a Siemens *D5000* applying Cu Kα radiation in θ–θ geometry was performed for sample characterization prior to the DSC measurements.

## Supporting Information Available

SI Table 1 covers characteristic temperatures and transformation enthalpies of the ice I_c_ to ice I_h_ transition for each individual run at the indicated heating rates. SI Table 2 contains information on the relaxation exotherm prior to the polytypic ice I_c_ to I_h_ transformation (integration limits *T*_initial_/*T*_final_ and peak minimum *T*_min_, as well as the enthalpy change Δ*H*_relax_). SI Figure 1 depicts a typical heating scan prior to baseline correction and the applied baseline for reference. SI Figure 2 shows a comparison of (baseline corrected) DSC scans at heating rates 10, 30, and 50 K min^–1^, respectively, pointing out a shift of *T*_onset_ to higher temperatures with increasing heating rate. SI Figure 3 contains two heating scans (10 and 30 K min^–1^, respectively) starting from ice XVII (PDF)
Transparent Peer Review report available (PDF)
