# Supplementary material for: Enthalpy Change from Pure Cubic Ice Ic to Hexagonal Ice Ih
Source: J Phys Chem Lett. 2023 May 25;14(21):5055–60. doi: 10.1021/acs.jpclett.3c00408 (PMC10240532; doi:10.1021/acs.jpclett.3c00408)
Supplement: Supplementary file 1 — jz3c00408_si_001.pdf [file jz3c00408_si_001.pdf]

Enthalpy Change from Pure Cubic Ice I_c to Hexagonal Ice I_h

Christina M. Tonauer [1], Keishiro Yamashita [1], Leonardo del Rosso [2], Milva Celli [2], Thomas Loerting [1,*]

[1] *Institute of Physical Chemistry, University of Innsbruck, Innrain 52c, A-6020 Innsbruck, Austria*

[2] *Consiglio Nazionale delle Ricerche, Istituto di Fisica Applicata 'Nello Carrara', via Madonna del Piano 10, I-50019 Sesto Fiorentino, Italy*

* e-mail: thomas.loerting@uibk.ac.at

Supporting Information (SI)

SI Tab. 1: Measured temperatures and transformation enthalpies of the ice I_c to ice I_h transition for each individual run at the indicated heating rates. T_{onset} and T_{offset} were extracted from the thermogram as indicated in Fig.1a. That is, they are the temperature at the intersection of the extrapolated baseline before (after) the peak occurs with the extrapolated low (high) temperature peak edge, resulting T_{onset} (T_{offset}). $T_{initial}$ (T_{final}) mark the integration limits for determining $\Delta H_{c \rightarrow h}$ and were drawn at the position where the exothermic peak first arises from (returns to) the baseline before (after) the exothermic event. T_{min} denotes the temperature at the peak minimum. "STDEV.S" represents the standard deviation based on a sample of the population.

	$T_{initial}$/K	T_{onset}/K	T_{offset}/K	T_{final}/K	T_{min}/K	$\Delta H_{c \rightarrow h}$ /(J mol^{-1})
#1_10Kmin^{-1}	214.0	219.9	229.0	231.3	225.2	39.5
#2_10Kmin^{-1}	211.0	220.7	229.4	231.0	225.7	37.8
AVERAGE (10Kmin^{-1})	**212.5**	**220.3**	**229.2**	**231.1**	**225.5**	**38.6**
STDEV.S (10 Kmin^{-1})	**2.1**	**0.5**	**0.3**	**0.2**	**0.4**	**1.2**
#1_30Kmin^{-1}	219.8	226.3	236.0	239.4	231.1	41.6
#2_30Kmin^{-1}	220.9	226.5	235.0	240.0	231.0	38.8
#3_30Kmin^{-1}	224.2	225.9	235.3	240.2	231.0	34.0
#4_30Kmin^{-1}	220.0	226.1	235.4	240.0	231.0	38.2
AVERAGE (30Kmin^{-1})	**221.2**	**226.2**	**235.4**	**239.9**	**231.0**	**38.2**
STABW.S (30Kmin^{-1})	**2.0**	**0.2**	**0.4**	**0.4**	**0.1**	**3.1**
#1_50Kmin^{-1}	224.3	230.3	239.8	242.7	235.0	35.3
#2_50Kmin^{-1}	224.7	229.9	238.9	241.5	234.8	38.2
#3_50Kmin^{-1}	223.8	229.6	239.1	242.4	234.6	36.2
AVERAGE (50Kmin^{-1})	**224.3**	**229.9**	**239.3**	**242.2**	**234.8**	**36.6**
STDEV.S (50 Kmin^{-1})	**0.5**	**0.4**	**0.4**	**0.6**	**0.2**	**1.5**
AVERAGE (all #)	220.3	226.1	235.3	238.7	231.0	**37.7**
STDEV.S (all #)	4.9	3.7	3.9	4.4	3.6	**2.3**

SI Tab. 2: the same as SI Tab.1, but for the relaxation exotherm (prior to the ice I_c to I_h transition) centered around ~193 K.

#ID	$T_{initial}$/K	T_{final}/K	T_{min}/K	ΔH_{relax}/(J mol^{-1})
#1_10Kmin^{-1}	168	209	186	17
#2_10Kmin^{-1}	xx	xx	xx	xx
AVERAGE (10Kmin^{-1})	**168**	**209**	**186**	**17**
STDEV.S (10Kmin^{-1})	xx	xx	xx	xx
#1_30Kmin^{-1}	168.8	219.8	193.2	13.8
#2_30Kmin^{-1}	169.7	220.9	195.4	14.8
#3_30Kmin^{-1}	171.3	221.7	195.0	17.8
#4_30Kmin^{-1}	170.9	218.9	189.9	11.8
AVERAGE (30Kmin^{-1})	**170.2**	**220.3**	**193.4**	**14.5**
STABW.S (30Kmin^{-1})	**1.2**	**1.2**	**2.5**	**2.5**
#1_50Kmin^{-1}	xx	xx	xx	xx
#2_50Kmin^{-1}	172.8	222.2	196.8	11.9
#3_50Kmin^{-1}	172.0	222.9	195.9	14.1
AVERAGE (50Kmin^{-1})	**172.4**	**222.5**	**196.3**	**13.0**
STDEV.S (50Kmin^{-1})	**0.5**	**0.5**	**0.7**	**1.6**
AVERAGE (all #)	170.5	219.3	193.2	**14.4**
STDEV.S (all #)	1.6	4.9	3.8	**2.3**

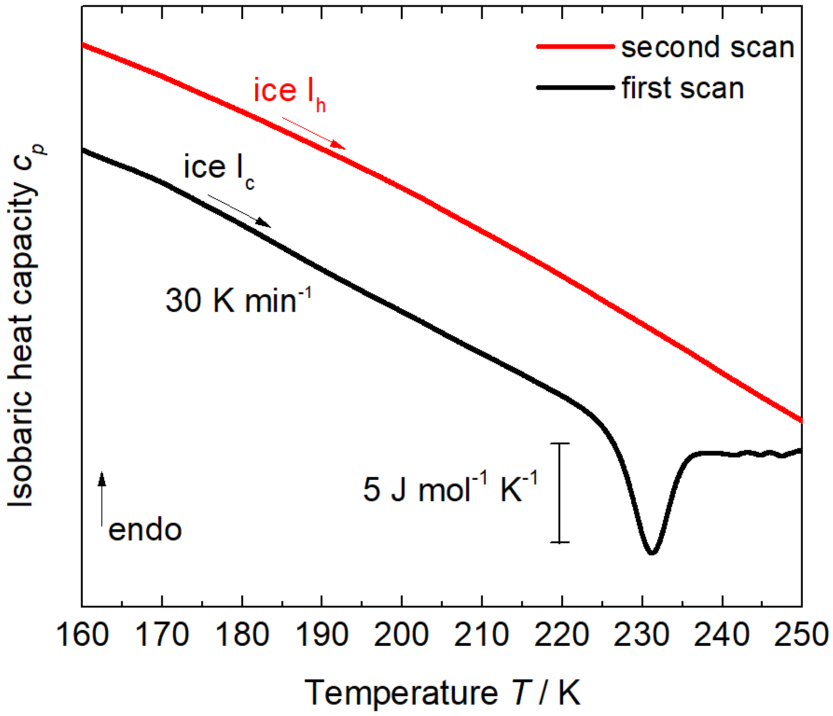

SI Fig. 1: A typical, "raw" heating scan at of an ice I_c sample performed at 30 K min^{-1} (black). For baseline correction the sample was recooled to 93 K after complete transformation to ice I_h and subsequently heated in a second scan (red). This second heating scan was subtracted from the first one for each measured sample.

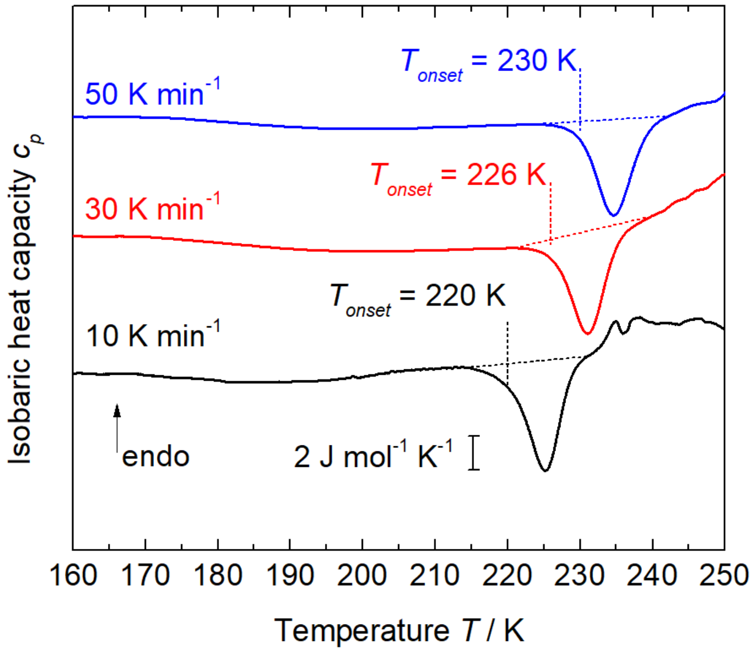

SI Fig. 2: Comparison of (baseline corrected) heating scans of ice I_c at 10 K min^{-1} (black), 30 K min^{-1} (red) and 50 K min^{-1} (blue). The shift of the I_c - I_h exotherm to higher temperatures with increasing heating rate is indicated by the increasing onset temperatures (marked by intersection of dashed lines).

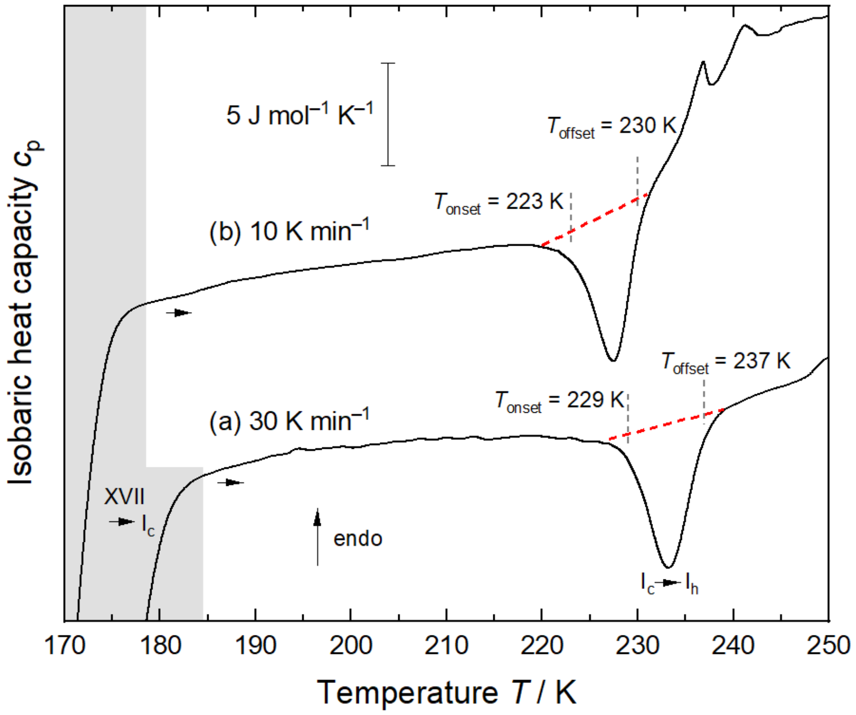

SI Fig. 3: Comparison of two baseline-corrected DSC scans heated at **(a)** 30 K min^{-1} and **(b)** 10 K min^{-1}, starting from ice XVII, cold loaded at 93 K. The ice XVII to ice I_c transition ceases ~ around 175 K, depending on the heating rate (highlighted in grey). The onset and offset temperatures of the ice I_c to I_h transition are marked by vertical dashed lines. Compared with DSC scans starting from ice I_c, the onset temperatures of the I_c-I_h transition are ~3 K higher if the starting material is ice XVII (see SI Tab. 2). The integration ranges of the respective transition exotherm are indicated by red dashed lines. Note the absence of the relaxation exotherm in these scans.
