# Supplementary material for: Enthalpy Change from Pure Cubic Ice Ic to Hexagonal Ice Ih
Source: J Phys Chem Lett. 2023 May 25;14(21):5055–60. doi: 10.1021/acs.jpclett.3c00408 (PMC10240532; doi:10.1021/acs.jpclett.3c00408)
Supplement: Supplementary file 2 — jz3c00408_si_002.pdf [file jz3c00408_si_002.pdf]

jz-2023-00408v.R1

Name: Peer Review Information for "Enthalpy Change from Pure Cubic Ice $I_c$ to Hexagonal Ice $I_h$"

First Round of Reviewer Comments

Reviewer: 1

Comments to the Author

This manuscript describes a calorimetric study on the transition from pure cubic ice to hexagonal ice. The advance made in this study is that cubic ice without hexagonal stacking faults has been employed in this study. The authors followed the recipe of references 32 and 33 in the manuscript to achieve this for their samples. This is the main and important contribution made in this study. The authors find that the enthalpy change from cubic ice to hexagonal ice is -37.7 joules mole-1. This is a very detailed and careful study. My comments are therefore minor ones intended to help the authors clarify points in the manuscript.

• The role of proton disorder should be mentioned since this perhaps can play a significant role in the values obtained. Does the proton disorder remain unchanged?

• On Pg. 7: the sentence with “highest possible vapor-pressure of cubic ice in our atmosphere.” should be more specific to the temperature for example.

• Pg.8; C2 needs better definition. Also, the amount of hydrogen in the hydrate can vary. This should probably be emphasized

• On Pg.8: the statement “- - -melting enthalpy of Ih " should be - - - melting enthalpy of ice Ih..

In summary, this manuscript provides a clear detailed report of the enthalpy change for the transformation from cubic ice to hexagonal ice. It is also clear that it will attract considerable discussion since the value reported is for cubic ice prepared under one particular condition that may influence the proton order in the cubic ice that could not be characterized by X-ray diffraction.

Reviewer: 2

Comments to the Author

Unfortunately, I have concerns regarding this study and the validity of its conclusions:

* The calorimetry data is complex and various processes seem to overlap. For example, looking at the data in Figure 1(a), the integrations limits could be chosen from 170 to 240 K to integrate over the two overlapping processes. The current integration may therefore significantly underestimate the enthalpy change related to the 2nd feature. But also, the slope after the 2nd feature is very steep. Looking at

Figure 1 in the SI, it is clear that something is happening after the 2nd exotherm which is not seen for pure ice Ih (which the sample should be at this stage according to the interpretation of the authors). On the basis of the current data, it is not clear how the transition enthalpy of bulk ice Ic to ice Ih can be determined reliably.

* Even if an ice Ic to ice Ih feature would be base-line separated from other processes, the transition enthalpy may still not reflect the ice Ic to ice Ih phase transition of the bulk materials. The phase transition is irreversible. So additional contributions from surface reductions, strain effects or defect elimination can never be excluded. This is a particular concern since the reported energy values are very small.

* Since the phase transition is irreversible, it is not possible to use the Clausius Clapeyron equation which requires delta_H from a reversible phase transition. Vapor pressure differences should hence not be calculated from this data.

* There are weak Bragg peaks related to ice Ih left and right of the cubic 111 peak. It does not seem as if the sample is really 100% cubic.

Reviewer: 3

Comments to the Author

Ice I can exist in two pure polymorphs, ices Ic and Ih, as well as polytypes in which there are variable number of stacking faults (the latter mistakenly called “cubic” ice until recently). Questions about the difference in free energy, enthalpy and entropy between these ice I forms have been pervasive for decades. The enthalpy of the “cubic” to hexagonal transition, in particular, has been reported in multiple experiments using with “cubic” ices obtained by multiple routes. The results have been quite variable, probably because the materials were not cubic ice and they had different structures. Only recently, methods that make almost pure cubic ice (with very little hexagonal faults) have been reported, opening the possibility of obtaining more accurate enthalpies of the cubic to hexagonal transition. This paper realizes this possibility, reporting calorimetric data for the cubic to hexagonal ice transition starting with ~97% pure cubic ice made by heating ice XII to 150 K at ambient pressure. The authors demonstrate that the heating rate impacts the pre-transformation peak that they attribute to consolidation and removal of defects and interfaces of the cubic particles, but does not impact the enthalpy of the annealed cubic ice to hexagonal ice transition, for which they report -37.7 +-2.3 J/mol at ~230 K. The value reported here is essentially the same as some previous reports (see below) but is accompanied by more certainty on the almost-pure cubic ice nature of the initial phase. Because of that, I think this is an important result that deserves publication. However, I have concerns about the presentation/contextualization and discussion of the paper that refrain me from recommending publication of the manuscript in its current form. I explain my concerns in what follows.

1) The authors frame the relevance of this study in terms of its implications for the vapor pressure of atmospheric ice, making the claim that cubic ice is common in the coldest regions of Earth’s atmosphere and using in eq. 1 the approximation that the free energy difference between the ices Ic and Ih is the enthalpy difference. However, this rationale for the significance of the paper has several issues:

a) there are no evidences for the existence of pure cubic ice in the atmosphere (where the ice is formed from supercooled water, amorphous solid water or vapor, and not from high pressure ice phases as in the lab experiments that produce the more pure cubic ices).

b) the vapor pressure of such atmospheric ices have been measured in experiments, so there is no need to reconcile the results using pure cubic ice … nor does the paper discuss such a reconciliation.

c) the enthalpy is not the free energy, and there are already experimental bounds to the free energy difference between cubic and hexagonal ice, and the value is lower than the enthalpy difference found in this study. The experimental free energy of annihilation of a single stacking fault in ice is 0.31± 0.03 $mJ/m^2$ (Hondoh et al "Formation and annihilation of stacking faults in pure ice" J. Phys. Chem. 1983). Using the unit cell dimensions of ice, this translates into a free energy cost of 16.5 ± 1.7 J/mol of water in the cubic layer (see conversion in Lupi et al. "Role of stacking disorder in ice nucleation" Nature 2017, ref. 8 of this manuscript). The cost of the stacking fault includes not only the excess free energy of cubic vs hexagonal ices, but also the cost of the two cubic-hexagonal interfaces. Free energy calculations with the mW water model (Hudait et al. "Free energy contributions and structural characterization of stacking disordered ices" PCCP 2016) predict that the cost of the cubic layer in hexagonal ice is 15.3 ± 2.3 J/mol, or which only 2.0 ± 1.5 J/mol corresponds to the excess free energy of cubic over hexagonal ice, and the rest to the the cost of the hexagonal-cubic interfaces (the mW model, for reference, predicts that the enthalpy difference between cubic and hexagonal ice is 0 ± 30 J/mol (Moore et al. "Freezing, melting and structure of ice in a hydrophilic nanopore", PCCP 2010). Thus, there is already an upper bound for the free energy difference between cubic and hexagonal ice (16 J/mol), a modeling study that reproduces it and indicates that the cubic-hexagonal bulk difference is even smaller, and these results are lower than the enthalpy. Overall, these results seem to indicate that cubic ice is destabilized by enthalpy but stabilized by entropy, with $\Delta S$ about 0.18 J/Kmol in favor of cubic ice at 200 K.

I do not intend with this to detract from the importance of knowing the enthalpy, but to emphasize that its significance for predicting the vapor pressure of atmospheric ice is not justified, and also that the authors could and should compare with the (scarce but accurate) data currently available for the stability of cubic vs hexagonal ice.

I would also beg the authors to change the way they introduce results as if they were for ice Ic without mentioning that most if not all of these previous "cubic" ices are actually stacking disordered ices and not cubic. It is mortifying for me to find that a paper that deals with real cubic ice propagates that misconception/confusion in its introduction. I recommend the authors to reframe the introduction in terms of the debate about the value of the enthalpy of the transformation to ice Ih for ices previously identified as cubic, without making the vapor pressure the central argument – but may be the more fundamental question of how different is the thermodynamics of these two so closely related ice I polymorphs. I think that is a fundamental question worth of discussion in JPC Letters.

2) The manuscript does a poor job at framing the results of this work with previous ones in the literature. This is both through misinterpretation and omission.

a) In terms of misinterpretation, I highlight here the mistaken claims in the first paragraph that recent simulation studies show that nanosize ice Ic is favored over nanosized ice Ih in terms of free energy.

To my knowledge, no water model predicts cubic ice to be more stable than hexagonal ice. TIP4P, TIP4P/Ice and ST2 predict free energies of cubic and hexagonal ice indistinguishable within the error bar of about 9 J/mol (see an account of what is known for the thermodynamics of stacking disordered ices in the Methods subsection “Thermodynamics of stacking faults in ice” of Lupi et al. Nature 2017), while mW predicts cubic ice to be 2.0 ± 1.5 J/mol less stable than hexagonal ice at 200 K.

All studied water models indicate that stacking disordered ice forms at the the nanoscale, none shows the result indicated in this manuscript of ice Ic nuclei. Only Lupi et al. Nature 2017 computes the free energy of the cubic, hexagonal and stacking disordered forms of the ice nuclei, finding that cubic is less stable than hexagonal and both are less stable than stacking disordered ice, which is there explained in terms of the entropic gain of the stacking disorder. The other articles cited in that claim about nanosize ice do not compute the free energy of cubic, hexagonal and stacking disorder (or even cubic and hexagonal nuclei) so they cannot make the claim about their relative stability.

Moreover, there is no size dependence discussion of the nucleation mechanism in ref. 7, that studies the nucleation at a single temperature and does not analyze the thermodynamics of the nuclei at various sizes (only their structure as they grow at 230 K).

In summary, the first paragraph of the manuscript misrepresents the simulation studies and their findings. I recommend the authors to read more carefully these papers, and correct the misstatements.

b) In terms of omission, the authors should elaborate more on the agreement with previous measurements. In particular, to those that predicted almost the same enthalpy for the transition. I am taking the values and refs here from the Methods section on thermodynamics of stacking disorder in Lupi et al. Nature 2017 (the enthalpies are shown as excess of cubic over hexagonal), that has a more detailed discussion of the trends in the experimental enthalpies of transformation according to the origin of the cubic ice:

“In ref. 41, ‘cubic’ ice was produced through decompression of ice IV and ice XII prepared by isobaric heating of high density amorphous ices at high pressures. Heating of cubic ice led to hexagonal ice with an enthalpy of transformation, measured by differential scanning calorimetry, of $\Delta H = 20 \pm 7$ J mol −1 (for ice IV at 216 K) and $\Delta H = 31 \pm 3$ J mol −1 (for ice XII at 217 K).”

where Ref 41 is: Salzmann, C.G., Mayer, E. and Hallbrucker, A., 2004. “Thermal properties of metastable ices IV and XII: Comparison, isotope effects and relative stabilities”. Physical Chemistry Chemical Physics, 6(6), pp.1269-1276.

“In ref. 39, ‘cubic’ ice was produced through decompression of ices III and IX prepared directly from water at high pressure (at 12–160 K). The enthalpy of trans- formation of stacking-disordered ice to hexagonal ice was obtained by calorimetry to be $\Delta H = 35.6 \pm 0.8$ J mol −1 (at 200 K)”

where Ref 39 is Yamamuro, O., Oguni, M., Matsuo, T. & Suga, H. “Heat capacity and glass transition of pure and doped cubic ices”. J. Phys. Chem. Solids 48, 935–942 (1987).

Salzmann’s paper is not cited in this manuscript. Yamamuro’s paper is cited as ref. 19 but is not discussed and the agreement of the enthalpy difference is not discussed. It is interesting to note that in these experiments the cubic ices were obtained from different high-pressure ice forms. A discussion of the

structure of these cubic ices –as available from these and other papers, and their level of purity, would be important for the discussion and context of the result of this work.

c) I recommend that the authors read/cite the work of Takeo Hondoh on the annihilation of stacking faults (cited above) as measure of stability of cubic vs hexagonal ice, and also his studies of the mechanism of elimination of stacking faults “Dislocation mechanism for transformation between cubic ice Ic and hexagonal ice Ih” Phil. Mag. 2015 that explains why the presence of small amount of hexagonal layers promotes the transition to hexagonal ice. I think these studies will help contextualize the findings and implications of the present work.

Author's Response to Peer Review Comments:

#The page numbers we refer to are the ones in the revised marked manuscript. (In the "clean" version, we have eliminated one page).

#We also have added the following sentences to the Acknowledgement section:

"The authors thank Andrea Donati (IFAC-CNR) for the technical support. M. C. and L. d. R. aknowledge the support from the Fondazione Cassa di Risparmio di Firenze under the contract "Grandi Attrezzature 2019 - HYDRO10000" (2019/0244)."

---------------

Reviewer: #1

*Comments: This manuscript describes a calorimetric study on the transition from pure cubic ice to hexagonal ice. The advance made in this study is that cubic ice without hexagonal stacking faults has been employed in this study. The authors followed the recipe of references 32 and 33 in the manuscript to achieve this for their samples. This is the main and important contribution made in this study. The authors find that the enthalpy change from cubic ice to hexagonal ice is -37.7 joules mole$^{-1}$. This is a very detailed and careful study. My comments are therefore minor ones intended to help the authors clarify points in the manuscript.*

• *The role of proton disorder should be mentioned since this perhaps can play a significant role in the values obtained. Does the proton disorder remain unchanged?*

Response: We thank Reviewer #1 for this remark. Tajima *et al.* [*Nature*, 1982, **299**, 810-812] showed that (KOH doped) H-ordered ice XI disorders at 72 K. Even though the experimental preparation of a H-ordered counterpart for pure ice $I_c$ is still missing, ordering/disordering temperatures are expected to be similar to the ice $I_h$/XI case [Geiger *et al.,* J. Phys. Chem. C, 2014, **118**, 10989-10997]. In the present study, the reported enthalpy change $\Delta H_{c\rightarrow h,}$ takes place at ~226 K, *i.e.*, more than 150 K higher than the ice XI/$I_h$ disordering temperature. Therefore, we can rule out a change of the disorder of both, ice $I_c$ and ice $I_h$. We emphasize this fact now in the revised version as follows:

Change: p. 7, 3rd paragraph: "We emphasize that the transition starts from hydrogen-disordered ice $I_c$ and ends in hydrogen-disordered ice $I_h$. That is, there is no entropy

difference between the two ices in terms of hydrogen order. Hydrogen ordering in ice I is of relevance only below 72 K [43].”

*• On Pg. 7: the sentence with “highest possible vapor-pressure of cubic ice in our atmosphere.” should be more specific to the temperature for example.*

Response: We thank Reviewer #1 for pointing this out and have rephrased the sentence to:

Change: p. 8, 3rd paragraph: “In terms of absolute vapor pressure of cubic ice, we can then use the vapor pressure reported for ice $I_h$ by Marti & Mauersberger [47] as well as Mauersberger & Krankowsky [48]. Adding the 3% and 2%, the absolute vapor pressure above ice $I_c$ calculates as $7.19 \cdot 10^{-4}$ Pa at 170 K and $10.2 \cdot 10^{-4}$ Pa at 230 K.”

*• Pg.8; C2 needs better definition. Also, the amount of hydrogen in the hydrate can vary. This should probably be emphasized*

Response: We thank Reviewer #1 for the suggestion. The marked sentence in the last paragraph on p.8 contains the information about the complete degassing of hydrogen as reported by Komatsu *et al.* [3] as follows:

Change: “For pure ice $I_c$ made from the $C_2$ structure of hydrogen hydrate $H_2$-$H_2O$ (by complete degassing of hydrogen, resulting in the empty cubic host structure as reported by Komatsu *et al.* [3]) a transition temperature above 240 K was found.”

*• On Pg.8: the statement “- - -melting enthalpy of Ih " should be - - - melting enthalpy of ice Ih..*

Response: Exactly, we have changed it accordingly.

Change: “Experimental Methods”, p.9, line 6:
“The calorimetric features were normalized by the melting enthalpy of ice $I_h$.”

*In summary, this manuscript provides a clear detailed report of the enthalpy change for the transformation from cubic ice to hexagonal ice. It is also clear that it will attract considerable discussion since the value reported is for cubic ice prepared under one particular condition that may influence the proton order in the cubic ice that could not be characterized by X-ray*

*diffraction.*

We thank Reviewer #1 for the constructive assessment of our manuscript.

Reviewer:#2

*Comments:*

*Unfortunately, I have concerns regarding this study and the validity of its conclusions:*
** The calorimetry data is complex and various processes seem to overlap. For example, looking at the data in Figure 1(a), the integrations limits could be chosen from 170 to 240 K to integrate over the two overlapping processes. The current integration may therefore significantly underestimate the enthalpy change related to the 2nd feature.*

Response: Based on Figure 1(a) and SI Fig. 3, the present study shows that the integration limits should in fact not be chosen from 170 to 240 K. By comparing the thermograms starting from pure ice $I_c$ (Fig 1(a)) and starting from ice XVII (SI Fig 3, transitioning inside the calorimeter to ice $I_c$), we find that in both cases the sharp exothermic feature indicative of the $I_c$-$I_h$ transition is observed at ~226 K, with the same value of heat release, but only the former case exhibits the broad pre-peak. This is clear evidence for our interpretation of the pre-peak being a result of a relaxation phenomenon and critically depending on the thermal history of a sample. Furthermore, this interpretation has already been established by Kohl *et al.* (2000) (see Tab.1), who studied the effect of isothermal annealing of ice $I_{sd}$ at temperatures just below the $I_{sd}$-$I_h$ transition. They found that annealing a sample of stacking-disordered ice for 5 min at 193 K leads to the disappearance of the pre-peak. Using powder x-ray diffraction they confirmed that this effect is not based on the transition to hexagonal ice during annealing (based on the absence of a sharp 101 reflex (25.8°)), but rather indicating grain growth of ice $I_{sd}$ (based on the sharpening of the 100 shoulder of ice $I_{sd}$ (22.19°). As pointed out in Tab. 1 in our revised version, many studies in the past have overestimated the $\Delta H_{c\rightarrow h}$ value by including the pre-peak to their integration. In our case, the pre-peak shows an enthalpy of -14.4 ± 2.3 J $mol^{-1}$ – which is of course provided in the manuscript (p. 7, 4th paragraph).

*But also, the slope after the 2nd feature is very steep. Looking at Figure 1 in the SI, it is clear that something is happening after the 2nd exotherm which is not seen for pure ice Ih (which the sample should be at this stage according to the interpretation of the authors). On the basis of the current data, it is not clear how the transition enthalpy of bulk ice Ic to ice Ih can be determined reliably.*

Response: The above-mentioned steep slope is a known artefact, sometimes observable due to slight variations of the gas flow in the calorimeter towards the end of a heating scan (here, close to 250 K) and can therefore be ignored.

** Even if an ice Ic to ice Ih feature would be base-line separated from other processes, the transition enthalpy may still not reflect the ice Ic to ice Ih phase transition of the bulk materials. The phase transition is irreversible. So additional contributions from surface reductions, strain effects or defect elimination can never be excluded. This is a particular concern since the reported energy values are very small.*

Response: As mentioned above, Kohl *et al.* (2000) show that the contributions from surface reductions, strain and/or defect elimination can in fact be removed by annealing at temperatures below the onset of the $I_c$-$I_h$. In our study the scan of ice XVII, in which pure cubic ice is produced *in situ* also allows to remove these additional contributions. In the scan for ice $I_c$ the additional contributions are well separated so that they can be removed by not integrating the pre-peak.

** Since the phase transition is irreversible, it is not possible to use the Clausius Clapeyron equation which requires delta_H from a reversible phase transition. Vapor pressure differences should hence not be calculated from this data.*

Response: We agree that, strictly speaking, the Clausius-Clapeyron equation applies to equilibrium conditions only. However, it is an approximation commonly used in literature also for transitions from weakly metastable to stable phases, e.g., the cubic-to-hexagonal ice transition (Shilling *et al.*, Geophys. Res. Lett, 33, 2006, **33**, L17801), (Murphy and Koop, Q. J. R. Meteorol. Soc.; 2005, **131,** 1539-1565 ). We also emphasize the fact that the equation is an approximation in the paragraph above equation 1 on page 8:

"Furthermore, this result can be applied for an approximation of the difference in vapor pressure between ice $I_c$ ($p_c$) and ice $I_h$ ($p_h$), a key question for supersaturation above ice clouds and the mechanism of cloud formation [49]. Based on the Clausius-Clapeyron equation, the ratio of the vapor pressures is often approximated [32] as:"

** There are weak Bragg peaks related to ice Ih left and right of the cubic 111 peak. It does not seem as if the sample is really 100% cubic.*

Response: We cannot observe a signal above noise level on the right of the 111 peak (a hypothetical 101 peak). On the left of the 111 peak, we do see a tiny peak and approximate a purity of 97 % for the cubic ice studied here. We address this as follows on p. 6 and 7, last and first paragraph, respectively:

"In our case, there is a tiny peak at this angle (23.00°), where the intensity is less than 3% of the 111 Bragg peak intensity. Other Bragg peaks of ice Ih are below the noise level of the measurement (positions marked by crossed grey arrows, taken from ref. [47]), which indicates that no hexagonal ice has condensed from air onto the sample. That is, there is a very small inherent amount of hexagonal stacking faults in our pure cubic ice. For comparison, "cubic ices" studied in the past prepared from other mother phases, *e.g.*, from high-pressure ice polymorphs, from amorphous ice or from the liquid, feature an intensity of the 100 Bragg peak of ice Ih at least an order of magnitude larger than seen in Figure 1b. This is why we follow the literature practice to call this ice "pure" ice $I_c$, essentially void of ice $I_h$."

For visualisation, we recommend comparison of X-ray patterns for differently prepared samples of ice $I_{sd}$, (as presented by Malkin *et al.* (PCCP, **2015**, 17, 60-76) in their Fig.3, reproduced below), with the X-ray pattern reported here in Fig.1b. The 111 Bragg peak (at 3.9 Å/23.0° in the Fig. below) is massive compared to the one reported in our study!

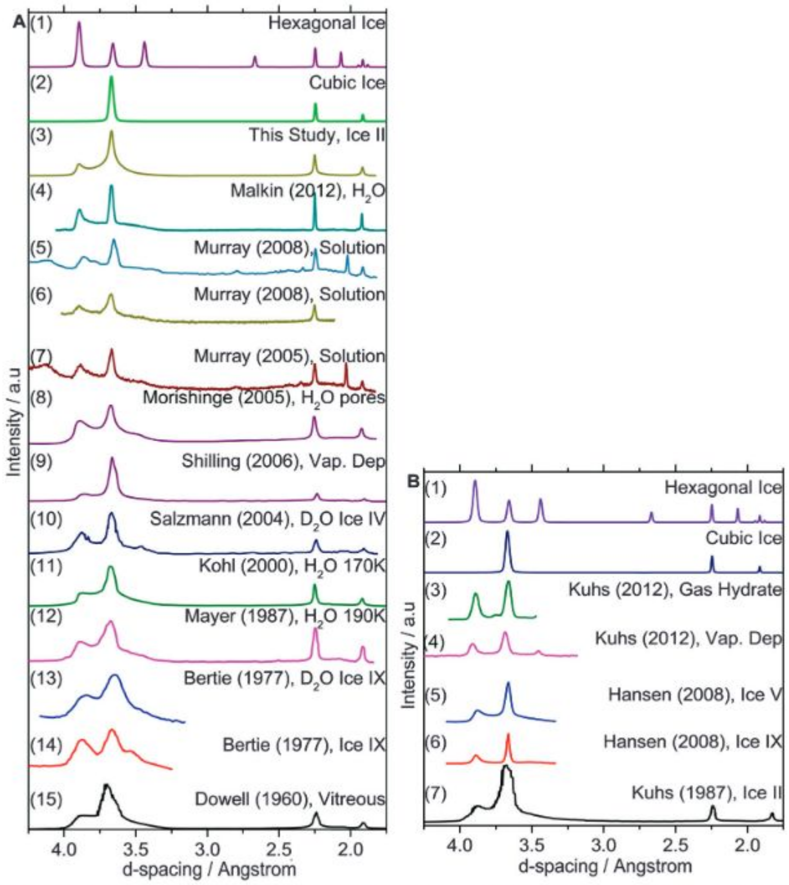


**Fig. 3** A selection of (A) X-ray and (B) neutron diffraction patterns of metastable ice I from the literature. Many of these patterns were digitised from the printed plots in the respective papers, and the quality of some of the older reproductions is relatively poor. Nevertheless, signatures of stacking disorder are apparent in all of these patterns, many of which were presented as ice $I_c$. The most obvious signatures of stacking disorder are the region between ~4 and 3.25 Å and the peak at ~3.8 Å. See details of each experiment and references in Table 1.

Reviewer:#3

*Comments: Ice I can exist in two pure polymorphs, ices $I_c$ and $I_h$, as well as polytypes in which there are variable number of stacking faults (the latter mistakenly called "cubic" ice until recently). Questions about the difference in free energy, enthalpy and entropy between these ice I forms have been pervasive for decades. The enthalpy of the "cubic" to hexagonal transition, in particular, has been reported in multiple experiments using with "cubic" ices obtained by multiple routes. The results have been quite variable, probably because the materials were not cubic ice and they had different structures. Only recently, methods that make almost pure cubic ice (with very little hexagonal faults) have been reported, opening the possibility of obtaining more accurate enthalpies of the cubic to hexagonal transition. This paper realizes this possibility, reporting calorimetric data for the cubic to hexagonal ice transition starting with ~97% pure cubic ice made by heating ice XVII to 150 K at ambient pressure. The authors demonstrate that the heating rate impacts the pre-transformation peak that they attribute to consolidation and removal of defects and interfaces of the cubic particles, but does not impact the enthalpy of the annealed cubic ice to hexagonal ice transition, for which they report -37.7 +-2.3 J/mol at ~230 K. The value reported here is*

*essentially the same as some previous reports (see below) but is accompanied by more certainty on the almost-pure cubic ice nature of the initial phase. Because of that, I think this is an important result that deserves publication. However, I have concerns about the presentation/contextualization and discussion of the paper that refrain me from recommending publication of the manuscript in its current form. I explain my concerns in what follows.*

Response: Thank you very much for the appreciation of our work and the recognition that the enthalpy of transformation measured here provides more certainty about the cubic-to-hexagonal transition than all previous calorimetric experiments.

*1) The authors frame the relevance of this study in terms of its implications for the vapor pressure of atmospheric ice, making the claim that cubic ice is common in the coldest regions of Earth's atmosphere and using in eq. 1 the approximation that the free energy difference between the ices Ic and Ih is the enthalpy difference. However, this rationale for the significance of the paper has several issues:*

*a) there are no evidences for the existence of pure cubic ice in the atmosphere (where the ice is formed from supercooled water, amorphous solid water or vapor, and not from high pressure ice phases as in the lab experiments that produce the more pure cubic ices).*

Response: We agree with Reviewer #3: there is no evidence for pure cubic ice in the atmosphere. The point we would like to make is simply the following: our approximation of the vapor pressure difference between cubic and hexagonal ice (using equ.1) marks an *upper limit* for the vapor pressure difference between the ice I polytypes present in the atmosphere, *i.e.*, ice $I_{sd}$ and ice $I_h$

*b) the vapor pressure of such atmospheric ices have been measured in experiments, so there is no need to reconcile the results using pure cubic ice … nor does the paper discuss such a reconciliation.*

Response: We think Reviewer #3 refers here to the vapor pressure measurements of Shilling *et al*. (Geophys. Res. Lett, 33, 2006, **33**, L17801). They in fact report a vapor pressure difference as large as 10.5 ±2.5 % between "cubic ice" (in fact ice $I_{sd}$) and ice $I_h$, while our approximation using $\Delta H_{c \rightarrow h}$ = -37.7 ± 2.3 J mol$^{-1}$ indicates a difference of only 3 % (170 K) and 2 % (230 K). For this reason we have some doubts about the results obtained by Shilling

*et al.* Because of this we want to include it in the manuscript. To emphasize this reasoning, we added the following sentence in the revised manuscript:

Change: p.8, 3th paragraph:

"That is, our result is inconsistent with measurements reporting a difference in vapor pressure of ~10 % between ice $I_{sd}$ and ice $I_h$ [32, 46], with the need of clarification and more direct measurements of the vapor pressure of ices $I_c$ and $I_{sd}$ in the future."

*c) the enthalpy is not the free energy, and there are already experimental bounds to the free energy difference between cubic and hexagonal ice, and the value is lower than the enthalpy difference found in this study. The experimental free energy of annihilation of a single stacking fault in ice is 0.31± 0.03 mJ/m2 (Hondoh et al "Formation and annihilation of stacking faults in pure ice" J.Phys. Chem. 1983). Using the unit cell dimensions of ice, this translates into a free energy cost of 16.5 ± 1.7 J/mol of water in the cubic layer (see conversion in Lupi et al. "Role of stacking disorder in ice nucleation" Nature 2017, ref. 8 of this manuscript). The cost of the stacking fault includes not only the excess free energy of cubic vs hexagonal ices, but also the cost of the two cubic-hexagonal interfaces. Free energy calculations with the mW water model (Hudait et al."Free energy contributions and structural characterization of stacking disordered ices" PCCP 2016) predict that the cost of the cubic layer in hexagonal ice is 15.3 ± 2.3 J/mol, or which only 2.0 ± 1.5 J/mol corresponds to the excess free energy of cubic over hexagonal ice, and the rest to the the cost of the hexagonal-cubic interfaces (the mW model, for reference, predicts that the enthalpy difference between cubic and hexagonal ice is 0 ± 30 J/mol (Moore et al. "Freezing, melting and structure of ice in a hydrophilic nanopore", PCCP 2010). Thus, there is already an upper bound for the free energy difference between cubic and hexagonal ice (16 J/mol), a modeling study that reproduces it and indicates that the cubic-hexagonal bulk difference is even smaller, and these results are lower than the enthalpy. Overall, these results seem to indicate that cubic ice is destabilized by enthalpy but stabilized by entropy, with ΔS about 0.18 J/Kmol in favor of cubic ice at 200 K.*

Response: We thank Reviewer #3 for providing these thermodynamic considerations. Trusting the mW model, we would agree with the conclusion about cubic ice being stabilized by entropy. However, the enthalpy obtained in our work (-37.7 ± 2.3 J $mol^{-1}$) is somewhat outside the predictions in the mW model (0 ± 30 J/mol), so we do not want to quantitatively

rely on the free energy difference of 2.0 ± 1.5 J/mol found for mW water. We thus solely report the enthalpy difference quantitatively and note the entropy difference qualitatively.

Change: We have added the following sentences to reflect this in the manuscript (p. 7, line 3 of 3rd paragraph):

"Our enthalpy difference compares with a calculated difference of 0±30 J $mol^{-1}$ in mW water [44]. Considering the free energy change $\Delta G_{c\rightarrow h}$ = -16.5 J $mol^{-1}$ measured by Hondoh [18, 19], our result implies that cubic ice is destabilized by enthalpy, but stabilized by entropy (which does not originate from Pauling entropy)."

*2 I do not intend with this to detract from the importance of knowing the enthalpy, but to emphasize that its significance for predicting the vapor pressure of atmospheric ice is not justified, and also that the authors could and should compare with the (scarce but accurate) data currently available for the stability of cubic vs hexagonal ice. I would also beg the authors to change the way they introduce results as if they were for ice Ic without mentioning that most if not all of these previous "cubic" ices are actually stacking disordered ices and not cubic. It is mortifying for me to find that a paper that deals with real cubic ice propagates that misconception/confusion in its introduction. I recommend the authors to reframe the introduction in terms of the debate about the value of the enthalpy of the transformation to ice Ih for ices previously identified as cubic, without making the vapor pressure the central argument – but may be the more fundamental question of how different is the thermodynamics of these two so closely related ice I polymorphs. I think that is a fundamental question worth of discussion in JPC Letters.*

Response: We thank Reviewer #3 for this remark and fully agree that the enthalpy difference between pure cubic ice and hexagonal ice (which is finally experimentally accessible) is the fundamental question targeted by our study, not vapor pressure. We have now rearranged the introduction devoting the first two paragraphs to the primary topic (thermodynamics of ice I polytypes), and only a few lines to the secondary aspect (implications for ice $I_{sd}$ in the atmosphere) We also exclude "atmospheric ice "from the Abstract in the revised version and focus on thermodynamics only.

Changes: Abstract and Introduction are rephrased to emphasize the thermodynamics aspects rather than implications for ice clouds.

*2) The manuscript does a poor job at framing the results of this work with previous ones in the literature. This is both through misinterpretation and omission.*

*a) In terms of misinterpretation, I highlight here the mistaken claims in the first paragraph that recent simulation studies show that nanosize ice Ic is favored over nanosized ice Ih in terms of free energy.To my knowledge, no water model predicts cubic ice to be more stable than hexagonal ice. TIP4P,TIP4P/Ice and ST2 predict free energies of cubic and hexagonal ice indistinguishable within the error bar of about 9 J/mol (see an account of what is known for the thermodynamics of stacking disordered ices in the Methods subsection "Thermodynamics of stacking faults in ice" of Lupi et al. Nature 2017), while*

*mW predicts cubic ice to be 2.0 ± 1.5 J/mol less stable than hexagonal ice at 200 K.*

*All studied water models indicate that stacking disordered ice forms at the the nanoscale, none shows the result indicated in this manuscript of ice Ic nuclei. Only Lupi et al. Nature 2017 computes the free energy of the cubic, hexagonal and stacking disordered forms of the ice nuclei, finding that cubic is less stable than hexagonal and both are less stable than stacking disordered ice, which is there explained in terms of the entropic gain of the stacking disorder. The other articles cited in that claim about nanosize ice do not compute the free energy of cubic, hexagonal and stacking disorder (or even cubic and hexagonal nuclei) so they cannot make the claim about their relative stability. Moreover, there is no size dependence discussion of the nucleation mechanism in ref. 7, that studies the*

*nucleation at a single temperature and does not analyze the thermodynamics of the nuclei at various sizes (only their structure as they grow at 230 K).*

*In summary, the first paragraph of the manuscript misrepresents the simulation studies and their findings. I recommend the authors to read more carefully these papers, and correct the misstatements.*

Response: We agree with this criticism and apologize for misrepresenting the current state of simulation studies.

Change: The updated Introduction now covers the aspects mentioned by Reviewer #3.

*b) In terms of omission, the authors should elaborate more on the agreement with previous measurements.*

*In particular, to those that predicted almost the same enthalpy for the transition. I am taking the values and refs here from the Methods section on thermodynamics of stacking disorder in Lupi et al. Nature 2017 (the enthalpies are shown as excess of cubic over hexagonal), that*

*has a more detailed discussion of the trends in the experimental enthalpies of transformation according to the origin of the cubic ice:*
*"In ref. 41, 'cubic' ice was produced through decompression of ice IV and ice XII prepared by isobaric heating of high density amorphous ices at high pressures. Heating of cubic ice led to hexagonal ice with an enthalpy of transformation, measured by differential scanning calorimetry, of ΔH = 20 ± 7 J mol −1 (for ice IV at 216 K) and ΔH = 31 ± 3 J mol −1 (for ice XII at 217 K). where Ref 41 is: Salzmann, C.G., Mayer, E. and Hallbrucker, A., 2004. "Thermal properties of metastable ices IV and XII: Comparison, isotope effects and relative stabilities". Physical Chemistry Chemical Physics, 6(6), pp.1269-1276. "In ref. 39, 'cubic' ice was produced through decompression of ices III and IX prepared directly from water at high pressure (at 12–160 K). The enthalpy of trans- formation of stacking-disordered ice to hexagonal ice was obtained by calorimetry to be ΔH = 35.6 ± 0.8 J mol −1 (at 200 K)" where Ref 39 is Yamamuro, O., Oguni, M., Matsuo, T. & Suga, H. "Heat capacity and glass transition of pure and doped cubic ices". J. Phys. Chem. Solids 48, 935–942 (1987). Salzmann's paper is not cited in this manuscript. Yamamuro's paper is cited as ref. 19 but is not discussed and the agreement of the enthalpy difference is not discussed. It is interesting to note that in these experiments the cubic ices were obtained from different high-pressure ice forms. A discussion of the structure of these cubic ices –as available from these and other papers, and their level of purity, would be important for the discussion and context of the result of this work.*

Response: We agree that the comparison with earlier calorimetry experiments (all on ice $I_{sd}$) was incomplete in our initial draft. We have now collected all calorimetry data and compiled it in Table 1. This Table then also highlights the issue about incorrect integration and merged peaks that contain contributions from relaxation at interfaces, etc. in addition to the bulk enthalpy change for the cubic to hexagonal transition.

Change:
We now include Tab. 1 (page 4), summarizing calorimetric studies from literature and discuss it in the main text:

p.4:

"**Table 1**: Comparison of calorimetry studies on the ice $I_{sd}$ to ice $I_h$ transition. $T_{init}$ and $T_{final}$ denote the initial and final temperature of a peak in a calorimetric scan, respectively, *i.e.*, the integration limits for determination of the enthalpy change. $T_{onset}$ and $T_{min}$ represent the

temperatures at the onset and the minimum of an exothermic feature, respectively, as shown in Fig. 1a. "Merged" implies that the pre-peak overlaps with the main peak for the ice $I_{sd}$ to ice $I_h$ transition. Based on the results of the present study, we mark enthalpy values that were likely overestimated in literature by (*)."

(see Table in manuscript)

p.7: 4th and 5th paragraph:

"That is, the pre-peak is related to the powdering of the sample, size of grains, etc., but unrelated to the inherent rearrangement of cubic to hexagonal stacking sequences.

Considering the literature data shown in Tab. 1, studies integrating a "merged" exothermic feature overestimate the heat release. That is, all enthalpies that are labelled "merged" in Tab.1 contain a contribution from relaxation that is not related to the bulk enthalpy for the cubic-to-hexagonal transition. The "true" $\Delta H_{c\rightarrow h}$(ice $I_{sd}$) in these studies is less negative, closer to zero, than the apparent $\Delta H_{c\rightarrow h}$(ice $I_{sd}$) reported by the authors. We mark the studies which likely overestimated the enthalpy change by (*) in Tab.1. For example,"

p.8: 1st paragraph:

Furthermore, Yamamuro *et al.* [29] likely reported a too large (negative) enthalpy change, even though their value (-37±1 $Jmol^{-1}$) coincides with the value presented here. However, in light of the present study, their integration limits were chosen too broadly. Considering their Fig. 12 of ref. [29], integration between ~ 200 K and ~230 K, excluding the pre-peak feature merged with the actual $I_{sd}$-$I_h$ transition exotherm, seems most accurate.

*c) I recommend that the authors read/cite the work of Takeo Hondoh on the annihilation of stacking faults*
*(cited above) as measure of stability of cubic vs hexagonal ice, and also his studies of the mechanism of elimination of stacking faults "Dislocation mechanism for transformation between cubic ice Ic and hexagonal ice Ih" Phil. Mag. 2015 that explains why the presence of small amount of hexagonal layers promotes the transition to hexagonal ice. I think these*

*studies will help contextualize the findings and implications of the present work.*

Response: We now include the above-mentioned work of Takeo Hondoh in the introduction. There is no evidence for or against the proposed 2-step dislocation mechanism in our work, so we refrain from discussing the crystallographic mechanism of the transition. We, however, do find the promotion through hexagonal layers, which shifts the transition exotherms in our calorigrams to lower temperature.

Change: p, 3, last sentence in paragraph:

Hondoh *et al.* [18, 19] approached this question by applied X-ray topography to study formation and annihilation of stacking faults and reported a free energy cost of 0.31 mJ $m^{-2}$ (16.5 J $mol^{-1}$ [6]) at 253 K, associated with the annihilation of a hexagonal stacking fault in ice $I_c$.

(p. 7, line 4 of 3rd paragraph):

Considering the free energy change $\Delta G_{c \rightarrow h}$ = -16.5 J $mol^{-1}$ measured by Hondoh [18, 19], our result implies that cubic ice is destabilized by enthalpy, but stabilized by entropy (which does not originate from Pauling entropy).

jz-2023-00408v.R2

Name: Peer Review Information for "Enthalpy Change from Pure Cubic Ice $I_c$ to Hexagonal Ice $I_h$"

Second Round of Reviewer Comments

Reviewer: 1

Comments to the Author

The authors have modified the manuscript following suggestions from the first review. This manuscript is now what can be considered as a definitive study for the determination of the enthalpy change for the transformation from the cubic form of ice to the hexagonal ice form. The authors have carefully improved on the recipe of earlier studies as well as given a very thorough and critical historical summary of previous studies. The methods employed for both the preparation and characterization of the ice phases are very clearly described. Although no new physics is presented, this manuscript will provide the best enthalpy value of the ice Ic to Ih transformation for further studies of ice transformations. It is recommended for publication in this journal.

Author's Response to Peer Review Comments:

Dear Prof Editor,

thank you very much for considering our manuscript for publication in JPCL - as requested we upload a "clean" version of the manuscript as well as Supporting Information with page numbers in the required format.

Best regards,

Thomas Loerting
